# Alterations of epigenetics and microRNAs in cancer and cancer stem cell

**DOI:** 10.3389/fgene.2014.00283

**Published:** 2014-08-19

**Authors:** Yoshimasa Saito

**Affiliations:** Division of Pharmacotherapeutics, Keio University Faculty of PharmacyTokyo, Japan

**Keywords:** epigenetics, microRNAs, cancer, cancer stem cells, methylation

## Alterations of DNA methylation and histone modification in cancer

Epigenetics is an acquired modification of methylation and/or acetylation of chromatin DNA or histone proteins, which regulates downstream gene expression. Epigenetic alterations can be induced by aging, chronic inflammation and viral infection. Aberrant DNA methylation and/or histone modification at the CpG island promoter may induce inactivation of tumor suppressor genes and play critical roles in the initiation and progression of human cancer. *In silico* analysis is essential to investigate putative genetic and epigenetic elements of tumor suppressor genes such as *Rb1* gene. This may contribute genetic and epigenetic information modulating tissue-specific transcripts and expression levels of genes (Hajjari et al., 2014). Genome-wide analysis of DNA methylation by BeadChip assay is quite useful to identify aberrantly methylated genes in human cancers. *HIST1H3J, POU4F2, SHOX2, PHKG2, TLX3*, and *HOXA7* were identified as aberrantly methylated genes in human papillary thyroid cancers by genome-wide analysis of DNA methylation. In addition, papillary thyroid cancers with preferential methylation were significantly associated with mutations of the BRAF/RAS oncogenes. These hypermethylated genes may constitute potential biomarkers for papillary thyroid cancer (Kikuchi et al., 2013).

In 2010, the International Human Epigenome Consortium (IHEC) was established to coordinate the production of reference maps of human epigenomes for key cellular states (www.ihec-epigenomes.net/). In order to gain substantial coverage of the human epigenome, the IHEC is planning to decipher at least 1000 epigenomes. These multilayer-omics analyses including genome, epigenome, transcriptome, proteome and metabolome are important for elucidating the molecular carcinogenesis and for exploring biomarkers and therapeutic targets for human cancers (Kanai and Arai, 2014).

## Dysregulation of microRNAs (miRNAs) by epigenetic alterations in cancer

miRNAs are a class of endogenous non-coding RNAs that play an important role in the regulation of several cellular, physiological and developmental processes. Aberrant miRNA expression is associated with many human diseases including cancer. Specific miRNAs are aberrantly expressed and play roles as tumor suppressors or oncogenes during carcinogenesis. Barrett's esophagus is considered to be a complication of gastroesophageal reflux disease and a precursor lesion of esophageal adenocarcinoma. Expression levels of *miR-221* and *miR-222* were increased when cultured esophageal epithelial cells were exposed to bile acids, which is one of the risk factors of esophageal adenocarcinoma. These miRNAs are known to specifically target p27Kip1, which inhibits the degradation of CDX2. Thus the degradation of CDX2 was enhanced by up-regulation of *miR-221* and *miR-222* on exposure of esophageal epithelial cells to bile acids (Matsuzaki and Suzuki, 2014).

Important tumor suppressor miRNAs are silenced by epigenetic alterations, resulting in activation of target oncogenes in human malignancies. But some oncogenic miRNAs such as *miR-196* family, *miR-200* family and *miR-519d* are reported to be up-regulated via DNA hypomethylation in various cancers. Histone modifications also play important roles in the dysregulation of miRNAs. Conversely, dysregulation of miRNAs such as *miR-152, miR-29* family and *miR-101* is related to epigenetic alterations through targeting chromatin-modifying factors including DNMT1, DNMT3A, DNMT3B, and EZH2 in cancer. Aberrant methylation of miRNA genes could be a potential biomarker for detecting cancer and predicting its outcome (Suzuki et al., 2013). Several miRNAs are dysregulated in lung cancers in response to DNA methylation and histone modification including methylation of histone H3 lysine 9 (H3K9) and H3K27. In lung cancer, several miRNAs such as *miR-9* and *miR-34* family are silenced by DNA methylation, whereas *miR-212* is silenced by methylation of H3K9 and H3K27 rather than DNA methylation (Watanabe and Takai, 2013).

## Alterations of epigenetics and miRNAs in cancer stem cell

Stem cells have an ability to perpetuate themselves through self-renewal and to generate mature cells of various tissues through differentiation. Accumulating evidence suggests that a subpopulation of cancer cells with distinct stem-like properties is responsible for tumor initiation, invasive growth, and metastasis formation, which is defined as cancer stem cells (CSCs). CSCs express specific cell surface markers including CD44, CD133, and EpCAM. Recently, a novel 3D culture method for stem cells called “organoid culture” has been developed. This culture method uses a serum-free medium that includes only identified growth factors such as R-spondin 1, EGF, and Noggin. R-spondin 1 is a ligand for Lgr5, which is a marker for intestinal stem cells and an essential factor to activate Wnt signal in intestinal crypts. Intestinal organoid culture enabled to expand normal or tumor epithelial cells *in vitro* with stem cell properties. This model will become a powerful research tool in clarifying the molecular pathogenesis and drug susceptibility of CSCs. Manipulation of cancer-related genes in stem cells may reveal the molecular mechanism underlying human carcinogenesis (Fujii and Sato, 2014). On the other hand, the role of mesenchymal stem cells (MSCs) in cancer development is still controversial. MSCs may promote tumor progression through immune modulation, but other tumor suppressive effects of MSCs have also been reported. Since systemically administered MSCs can be recruited and migrated toward tumors, the incorporation of engineered MSCs can be used as novel anti-tumor carriers for the development of tumor-targeted therapies (Yagi and Kitagawa, 2013).

miRNAs including *let-7* and *miR-34a* have been implicated in the regulation of CSC properties by suppression of their target genes such as HMGA2, RAS, NOTCH1, and CD44. The modulation of CSC gene expression by miRNAs could be a novel therapeutic strategy targeting CSCs (Takahashi et al., 2014). Glioblastomas show heterogeneous histological features, which are considered to be associated with the presence of glioma stem cells (GSCs). GSCs have an ability to self-renew and initiate the growth of gliomas and are resistant to conventional chemotherapies. The oncogenic miRNAs including *miR-17-92* cluster is involved in the regulation of GSC differentiation, apoptosis and proliferation by suppression of target genes such as CTGF. The tumor suppressor miRNAs including *miR-34a* is also dysregulated in GSCs. *miR-34a* directly inhibits the expression of c-Met, Notch-1 and Notch-2 and involved in the differentiation of GSCs. Long non-coding RNAs (lncRNAs) such as *MEG3* and *CRNDE* are also dysregulated in glioma tissues and may be associated with the stemness of glioma cells (Katsushima and Kondo, 2014).

### Conflict of interest statement

The author declares that the research was conducted in the absence of any commercial or financial relationships that could be construed as a potential conflict of interest.
